# Differential Phenotypes of Tissue-Infiltrating T Cells during Angiotensin II-Induced Hypertension in Mice

**DOI:** 10.1371/journal.pone.0114895

**Published:** 2014-12-11

**Authors:** Zihui Wei, Iresha Spizzo, Henry Diep, Grant R. Drummond, Robert E. Widdop, Antony Vinh

**Affiliations:** Department of Pharmacology, Monash University, Clayton, Victoria, Australia; INSERM, France

## Abstract

Hypertension remains the leading risk factor for cardiovascular disease (CVD). Experimental hypertension is associated with increased T cell infiltration into blood pressure-controlling organs, such as the aorta and kidney; importantly in absence of T cells of the adaptive immune system, experimental hypertension is significantly blunted. However, the function and phenotype of these T cell infiltrates remains speculative and undefined in the setting of hypertension. The current study compared T cell-derived cytokine and reactive oxygen species (ROS) production from normotensive and hypertensive mice. Splenic, blood, aortic, kidney and brain T cells were isolated from C57BL/6J mice following 14-day vehicle or angiotensin (Ang) II (0.7 mg/kg/day, s.c.) infusion. T cell infiltration was increased in aorta, kidney and brain from hypertensive mice. Cytokine analysis in stimulated T cells indicated an overall Th1 pro-inflammatory phenotype, but a similar proportion (flow cytometry) and quantity (cytometric bead array) of IFN-γ, TNF-α, IL-4 and IL-17 between vehicle- and Ang II- treated groups. Strikingly, elevated T cell-derived production of a chemokine, chemokine C-C motif ligand 2 (CCL2), was observed in aorta (∼6-fold) and kidney in response to Ang II, but not in brain, spleen or blood. Moreover, T cell-derived ROS production in aorta was elevated ∼3 -fold in Ang II-treated mice (n = 7; P<0.05). Ang II-induced hypertension does not affect the overall T cell cytokine profile, but enhanced T cell-derived ROS production and/or leukocyte recruitment due to elevated CCL2, and this effect may be further amplified with increased infiltration of T cells. We have identified a potential hypertension-specific T cell phenotype that may represent a functional contribution of T cells to the development of hypertension, and likely several other associated vascular disorders.

## Introduction

Hypertension is a common risk factor for cardiovascular disease and stroke, which are the major causes of morbidity and mortality in Western societies (W.H.O, 2013) [1]. While current anti-hypertensive therapies can maintain blood pressure homeostasis in some patients, surprisingly 10–15% of cases of human hypertension remain resistant to these therapies, whether used alone or in combination [2], [3]. Moreover, despite extensive research, the etiology of hypertension still remains unclear and novel approaches need to be developed to treat this condition. Recent studies have implicated inflammation and activation of the immune system in the development of hypertension [4]. It is now well defined that T cells are required for the development of hypertension, which infiltrate organs that control blood pressure such as the aorta and kidneys [5], [6]. However, the functional contribution of these infiltrating T cells to the local inflammatory response during hypertension remains speculative and understudied.

T lymphocytes can be divided into several subtypes and subsets that all produce various responses to infection and immune homeostasis. The predominant subtypes are T helper (Th) cells (CD4+) and cytotoxic T cells (CD8+), but a population of double negative cells also exist (DN; CD4-CD8-). Approximately 95% of all T cells express a membrane-bound T cell receptor (TCR) comprised of α and β subunits, which is capable of recognizing specific antigens presented in the context of a major histocompatibility complex. A smaller population of T cells (5–10%) express a different TCR comprised of γ and δ subunits that recognize antigens that are usually not presented by MHC molecules. Antigen presenting cells such as dendritic cells and macrophages engulf foreign antigens and can present antigen-specific epitopes to T cells. In the presence of innate cytokines such as IL-12/IFN-γ, IL-4 and IL-23, Th cells (CD4+) polarise to Th1, Th2 and Th17 cells respectively [7]. Polarised Th subsets secrete adaptive immune cytokines that also include IFN-γ, TNF-α (both Th1), IL-4 (Th2) and IL-17 (Th17), which mount an immune response involving reciprocal activation of innate cells such as macrophages and eosinophils, as well as B cells of the adaptive immune system to remove the pathogen [7]. Cytotoxic T cells (CD8+) also act to kill pathogens by releasing cytotoxic enzymes [7]. DN cells lack the expression of both the surface proteins CD4 and CD8 and their functional role is still not completely understood [8]. The role of T cells in inflammatory diseases has been studied for decades, especially in autoimmune diseases such as rheumatoid arthritis [9] and systemic lupus erythematosus (SLE) [10]. Rheumatoid arthritis is known to be associated with infiltrating Th cells into synovial joints. As discussed above, Th cells are a large source of cytokines that can promote local inflammation as well as recruitment of other immune cells. In the setting of rheumatoid arthritis, pro-inflammatory cytokine levels are elevated, which includes IFN-γ, TNF-α, IL-6, IL-1, GM-CSF [11] and chemokines such as chemokine C-C motif ligand 2 (CCL2) [12] and CCL13 [13]. TNF-α has been documented to be of major importance in the pathogenesis of rheumatoid arthritis. However, there is also upregulation of homeostatic regulatory cytokines, IL-10 and TGF-β, which can suppress the pro-inflammatory response [9]. Importantly, research into T cells in rheumatoid arthritis has led to novel treatments to treat this disease such as the TNF-α inhibitor, etanercept [14] and IL-6 antagonist tocilizumab [15] which inhibit or neutralize TNF-α and IL-6 respectively.

A likely mechanism by which T cells may contribute to hypertension is through the release of cytokines, which promote inflammation in various blood pressure-controlling organs [4]. We have previously reported an elevation in Th1 activity in hypertensive mice shown by increased lymphoid Th1 specific cytokines IFN-γ and TNF-α [6], [16]. Shao and colleagues [17] also suggested that there are more Th1 cells in hypertensive rats. Although past studies have shown T cell-derived cytokines are associated with hypertension, they have focused on circulating or secondary lymphoid organ T cells in the setting of hypertension, [4], [6], [16] and have not studied the role of tissue-infiltrating T cells that represent local effects of T cells and inflammation. Moreover, although chemokines such as CCL2, and ROS production have been implicated in hypertension [6], [18], whether infiltrating T cells are a source of these influential mediators has not been examined in the setting of hypertension.

To our knowledge, we have conducted the first phenotypical analysis of aortic, renal and brain-infiltrating T cells from normotensive and hypertensive mice. After isolating T cells from various blood pressure-controlling organs we measured T cell-derived cytokine and ROS production. We report that organ-specific T cells from hypertensive mice exhibit phenotypically different profiles that may be responsible for local leukocyte recruitment and oxidative stress during hypertension.

## Materials and Methods

### Animals

Male C57BL6/J mice aged 10–12 weeks (n = 66–75) were obtained from Monash Animal Services (MAS) and housed on a 12 hour light/dark cycle with food and water provided *ad libitum*. Isoflurane-anaesthetized mice were randomly allocated to receive either vehicle (0.03M NaCl, 1% acetic acid) or Ang II (0.7 mg/kg/day) via osmotic minipumps (Alzet model 2002) implanted subcutaneously for 14 days. All surgical and treatment procedures were approved by the Monash Animal Research Platform Ethics Committee (Approval number MARP/2011/85).

### Blood Pressure Monitoring

Systolic blood pressures (SBP) were measured using a non-invasive tail-cuff apparatus (MC4000 Blood Pressure Analysis System, Hatteras Instruments) prior to treatment and on day 14 to confirm hypertension (SBP > 140 mmHg) was achieved. At each time point over 20 measurements were recorded and averaged.

### Tissue Harvesting & Preparation

At the end of each treatment period, mice were euthanized by carbon dioxide asphyxiation and blood and tissue were harvested for further analysis. This procedure was approved by the Monash Animal Research Platform Ethics Committee (Approval number MARP/2011/85). Cardiac puncture was employed to extract blood from the right ventricle. Mice were perfused using a perfusion pump (Model 77200-62 easy-load II, MasterFlex) with phosphate buffer solution (PBS; 137 mM NaCl, 2.7 mM KCl, 10 mM Na_2_HPO_4_, 1.76 mM KH_2_PO_4_) for five minutes. Spleen, kidneys, brain and aorta with perivascular fat intact were removed and maintained on ice.

Blood and spleen were prepared as previously described [6]. Aortic, brain and kidney samples were dissociated in 1.5 mL digestion buffer containing collagenase type XI (125 U/mL), hyaluronidase (600 U/mL) and collagenase type I-S (450 U/mL) with gentleMACS Octo Dissociator (Miltenyi Biotec) for 2 minutes and then digested at 37°C for 45 minutes. Following digestion, samples were pressed through a 70 µm filter and centrifuged at 1200 RPM for 10 minutes. Supernatants were then discarded and aortic samples were ready for further analysis. Kidney and brain samples were further subjected to percoll gradient centrifugation. Samples were resuspended in 2 mL of 40% (kidney) or 30% (brain) isotonic percoll solution (GE Healthcare Life Science) and then gently underlaid with 2 mL of 60% or 70% percoll solution respectively. Samples were then centrifuged at 2700 RPM with the centrifuge brake turned off for 25 minutes at room temperature. Following centrifugation, adipocytes and debris were aspirated off the top layer and mononuclear cells (MNCs) were collected at the interface of both solutions.

### T cell Enrichment

To isolate T cells from tissue samples, cell pellets were incubated with anti-CD90.2 labeled magnetic microbeads (Miltenyi Biotec). The cells were then isolated using a magnetic column as per manufactures instructions. Cell purity of enriched cells was confirmed to be >90% using flow cytometry. Following isolation, the cells were counted then resuspended in culture media and allocated for T cell-derived cytokine and ROS detection.

### Quantitative Cytokine and chemokine detection (Cytometric Bead Array)

Isolated T cells from various organs were seeded in 96-well plates coated with anti-CD3 antibody (BD PharMingen) and cultured for 48 hours in presence of anti-CD28 (BD Pharmingen) as previously described [16]. Following stimulation, conditioned media was used to analyze levels of IFN-γ, TNF-α, IL-4, IL-17 and CCL-2 using a cytometric bead array (CBA, BD Biosciences). To determine phenotypical differences between T cells from hypertensive and normotensive mice, cytokine amount was normalized to T cell number.

### Detection of T cell derived superoxide production

To detect the extracellular ROS produced from isolated T cells, L-012 chemiluminescence was employed. ROS formation was detected under basal conditions and following stimulation with the direct activator of protein kinase C, phorbol 12,13-dibutyrate (PDBu; 100 µM). Enriched cells were resuspended in Krebs-HEPES buffer (in mM: NaCl 118; KCl 4.7; KH_2_PO_4_ 1.2; MgSO_4_·7H_2_O 1.2; CaCl_2_ 2.5; NaHCO_3_ 25; glucose 11.7; HEPES 20, pH 7.4). Following a 30 minute incubation period, L-012 (100 mmol/L) was added to each well, which was then loaded into a Hidex Chameleon Luminescence detector. All samples were performed in triplicate and photon emissions were recorded for 30 cycles at 2 min intervals. Photon emissions (relative light units per second) were then averaged over the final 20 cycles.

To enumerate the proportion of ROS positive T cells and T cell subsets, intracellular ROS detection was performed in aortic samples that were harvested and digested, but not subjected to T cell enrichment as described above. Cell suspensions were stimulated with PDBu (100 µM) for 30 minutes at 37°C. Cells were then stained with a cell permeable ROS-sensitive dye, 5-(and-6)-chloromethyl-2′,7′-dichlorodihydrofluorescein diacetate, acetyl ester (CM-H_2_DCFDA; 1 µM) for a further 30 minutes at 37°C. Following incubation, cells were washed and stained with live/dead Aqua stain (Life Technologies) for 15 minutes at 4°C. After washing with FACS buffer (PBS with 0.5% bovine serum albumin) cells were stained with fluorochrome-conjugated antibodies for surface markers including CD45 (leukocytes; APC-Cy7; 30-F11), CD3 (T cells; APC; 145-2C11), CD4 (T-helper cells; BV605; RM4-5) and CD8 (cytotoxic T cells; PerCP-Cy5.5; 53–6.7). Cells were then analysed on a LSR II flow cytometer for (BD Biosciences). Unstimulated controls (minus PDBu) were also analysed to establish gates for ROS-producing T cells.

### Qualitative Intracellular Cytokine detection and Flow Cytometry

To identify the proportion of T cells and T cell subsets producing each cytokine, intracellular staining and flow cytometry was performed. In a subset of animals, processed tissues samples were not subjected to T cell enrichment and were stimulated with phorbol 12-myristate 13-acetate (PMA; 100 µg/ml)/ionomycin (1 µg/ml) for 4 hours at 37°C with 5% CO_2_ in the presence of protein transport inhibitors (Golgi plug/Golgi stop, BD Bioscience), which prevent the transport of cytokines out of the cell. Cells were washed and stained with live/dead stain (Life Technologies) and fluorochrome-conjugated antibodies for surface markers as described for intracellular ROS detection. Cells were then fixed and permeabilised using a Fix/Permeabilisation Buffer (eBiosciences) for intracellular staining of cytokines including IFN-γ(AlexaFluor700; XMG1.2), TNF-α (PE; TN3-19.12), IL-4 (PE-Cy7; 11B11), IL-17 (FITC; TC11-18H10.1). In a separate subset of samples, CCL2 (FITC; 2H5) was also stained to detect intracellular T cell-specific CCL2 production. Cells were then analysed using an LSR II flow cytometer (BD Biosciences). Negative unstimulated controls and fluorescence minus one (FMO) controls were analyzed to establish gates for cytokine-producing T cells.

### Statistical analyses

All data was expressed as mean ± standard error of mean (SEM). Changes in blood pressure were analyzed using two-way repeated measures ANOVA. Unpaired t-tests were used to compare vehicle and Ang II groups. A p-value of <0.05 was considered significant.

## Results

### Systolic Blood Pressure (SBP)

Using non-invasive tail-cuff measurements hypertension was confirmed in Ang II-treated mice (Day 14 SBP: vehicle 116±3 vs Ang II 158±2, P<0.0001).

### T cell Infiltration into Organs

**Figure 1:**
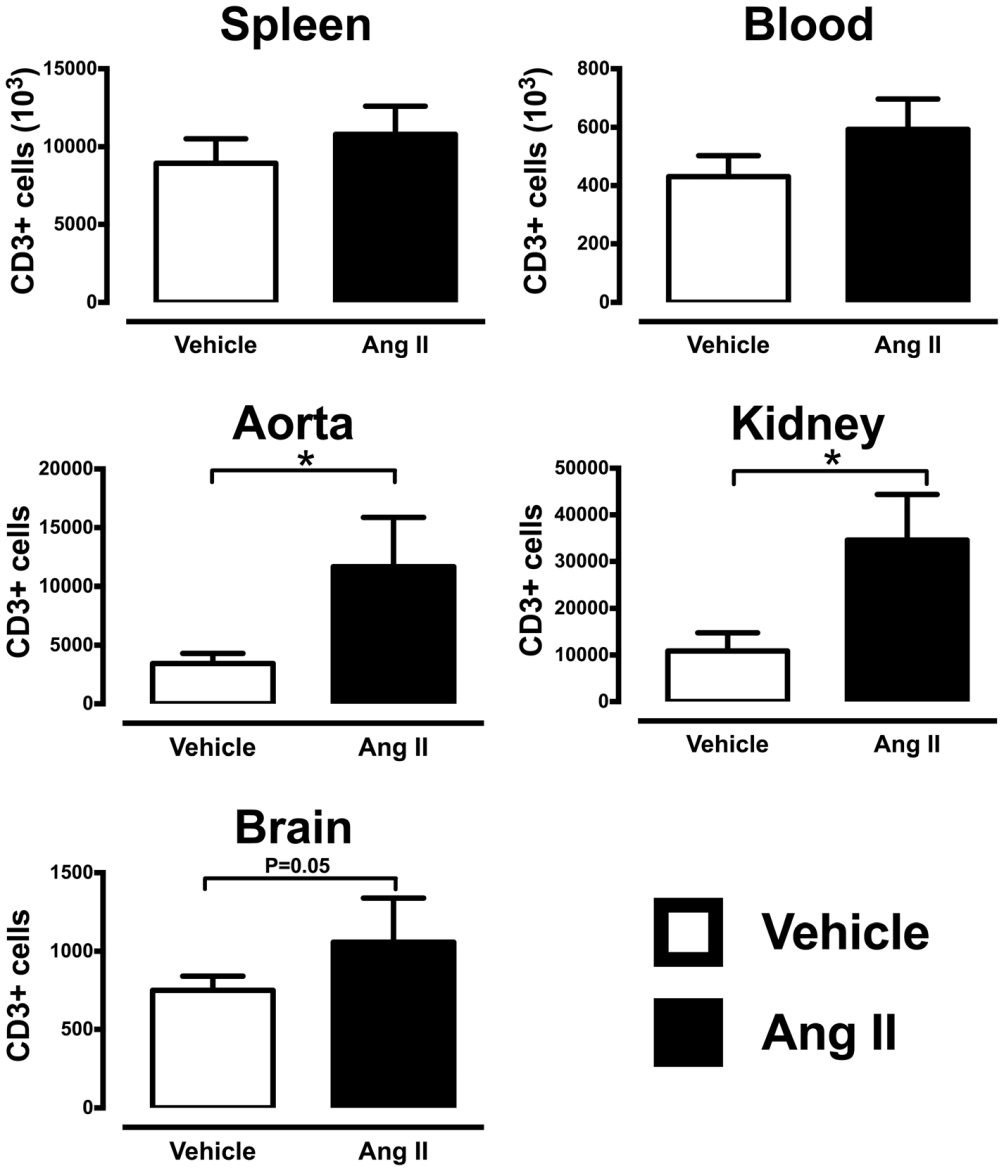
Total tissue infiltrating T cell (CD3+) infiltration into various organs. Flow cytometry was employed to count the number of T cells in vehicle- and Ang II treated mice in (A) non-infiltrating organs (blood and spleen) and (B) infiltrating organs kidney, brain and aorta. (*P<0.05 Vs vehicle; Unpaired t test; n = 12–19). (NB. Cell counts for kidney; brain and aorta were normalized to the cell counting beads).

Flow cytometry was used to quantify the number of T cells (CD3+) in various organs. Compared to vehicle-infused mice, Ang II-infusion did not alter the number of circulating or splenic T cells. However, Ang II-infusion did increase T cell numbers in the aorta, kidney and brain (Fig. 1). In the aorta and kidney, Ang II-infusion tended to increase all CD4+, CD8+ and DN T cell subsets compared to vehicle-treated mice (S1 Figure). However, only the aortic CD4+ and double negative T cells and kidney CD4+ T cells were of the effector phenotype (CD44^hi^+CD62L^lo^; S2 Figure). We also observed an increase in FoxP3+ T regulatory cell number in aorta, and a trend towards an increase in kidney from Ang II-infused mice (S3 Figure).

### Intracellular Cytokine Analysis

**Figure 2:**
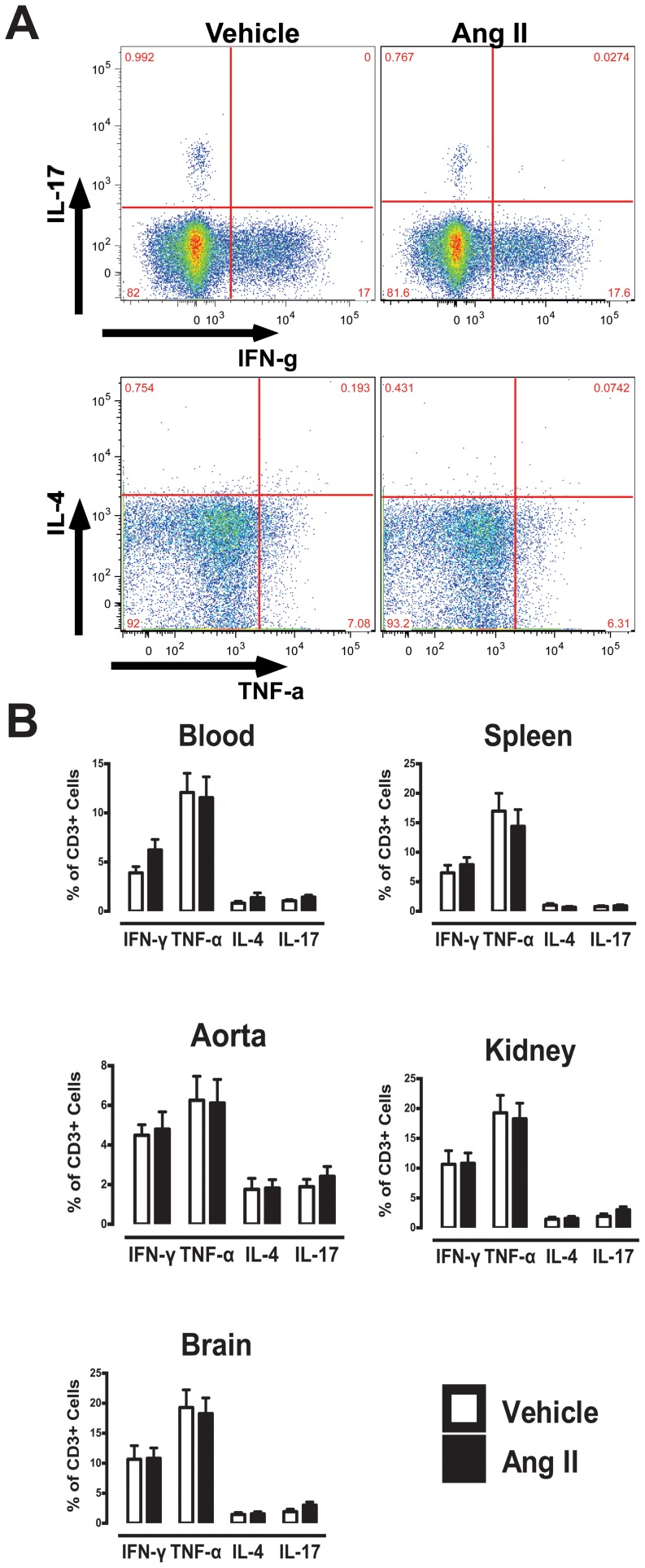
Intracellular FACS analysis of cytokine producing T cells (CD3+). (A) Representative flow cytometry plots and gating strategy of IFN-γ, TNF-α, IL-4 and IL-17-producing T cells in blood. (B) Mean data of cytokine-producing T cells in vehicle- and Ang II-treated group in various organs (n = 11–19).

Using intracellular staining and flow cytometric analysis, cytokine profiles of T cells in various tissues and organs was compared (Fig. 2). In T cells isolated from blood and all organs, a greater proportion of T cells produced typical Th1 cytokines (IFN-γ and TNF-α) relative to Th2 (IL-4) or Th17 (IL-17) (Fig. 2B). However, no significant differences in cytokine-producing T cells were observed between vehicle- and Ang II-treated mice.

Although no overall changes in Th1, Th2 and Th17 cytokines were observed in CD3+ cells, further analysis of organ-specific T cell subsets (CD4+, CD8+ and DN cells) revealed differences in cytokine production in certain subsets. Cytokine-producing T cell subsets from various organs of vehicle- or Ang II-treated mice revealed 3 unique subsets (Table 1). A significant increase in the proportion of aortic CD8+ T cells and kidney DN T cells that produced IFN-γ was observed in Ang II-treated mice while a trend towards greater proportion of IFN-γ-producing DN T cells in the blood of Ang II-treated mice was also observed (Table 1).

**Table 1:** Proportion of cytokine producing CD4+, CD8+ and DN T cell subsets in various organs from vehicle- and Ang II-infused mice.

		IFN-γ		TNF-α		IL-4		IL-17	
		Vehicle	Ang II	Vehicle	Ang II	Vehicle	Ang II	Vehicle	Ang II
**Blood**	**CD4**	2.0±0.3	1.7±0.2	14.6±2.8	16.5±2.7	0.5±0.1	0.4±0.1	0.7±0.2	1.1±0.2
	**CD8**	5.5±1.0	7.0 ±12	9.6±2.5	6.8±1.7	0.4±0.1	0.8±0.2	0.4±0.1	0.5±0.1
	**DN**	7.2 ±1.1	12.3 ±2.5	12.0±3.6	9.5±2.5	2.7±0.8	3.1±0.8	7.3±1.2	9.3±1.1
**Spleen**	**CD4**	3.2 ±0.6	3.4 ±0.3	20.3±3.7	21.3±3.8	1.6±0.1	0.6±0.2	0.6±0.1	0.7±0.2
	**CD8**	5.6 ±1.4	8.23±1.5	13.3±3.2	9.5±2.7	1.1±0.3	0.9±0.2	0.5±0.2	0.5±0.2
	**DN**	13.4 ±2.8	18.1 ±3.0	7.1±1.3	8.8±1.3	3.1±0.9	2.9±0.6	4.1±0.5	3.2±0.4
**Aorta**	**CD4**	7.4±1.5	6.1±1.4	8.7±2.3	12.9±2.8	0.7±0.2	1.2±0.4	0.8±0.3	1.1±0.3
	**CD8**	2.8±0.6	5.0±0.7 *	2.9±0.6	5.0±1.1	3.0±0.8	3.8±1.0	1.1±0.4	1.7±0.4
	**DN**	3.4±0.9	4.1±1.0	5.2±1.2	6.1±1.4	2.5±1.0	4.7±1.7	5.9±1.7	4.2±0.7
**Kidney**	**CD4**	5.7±2.2	9.9±2.6	3.6±0.7	3.7±1.1	2.0±0.5	1.7±0.5	1.7±0.3	1.5±0.4
	**CD8**	2.7±1.0	2.6±0.7	5.6±1.5	4.1±1.0	3.4±0.7	2.8±0.7	0.5±0.2	1.5±0.6
	**DN**	4.5±1.5	8.2±1.8 *	3.2±0.6	6.1±3.5	0.7±0.2	1.7±0.5	4.7±1.2	3.7±0.7
**Brain**	**CD4**	9.0±2.2	8.9±2.2	21.7±3.3	28.4±4.5	0.9±0.3	0.7±0.3	0.4±0.2	0.7±0.3
	**CD8**	11.7±3.1	11.4±2.3	16.4±3.7	13.7±2.6	1.2±0.4	2.9±0.8	1.9±0.6	2.3±0.7
	**DN**	9.5±2.7	14.4±3.2	12.7±3.8	7.4±2.4	2.7±1.5	5.4±1.9	8.7±3.4	8.1±3.7

*P<0.05 Vs Vehicle, Unpaired t –test, n = 11–19.

**Figure 3:**
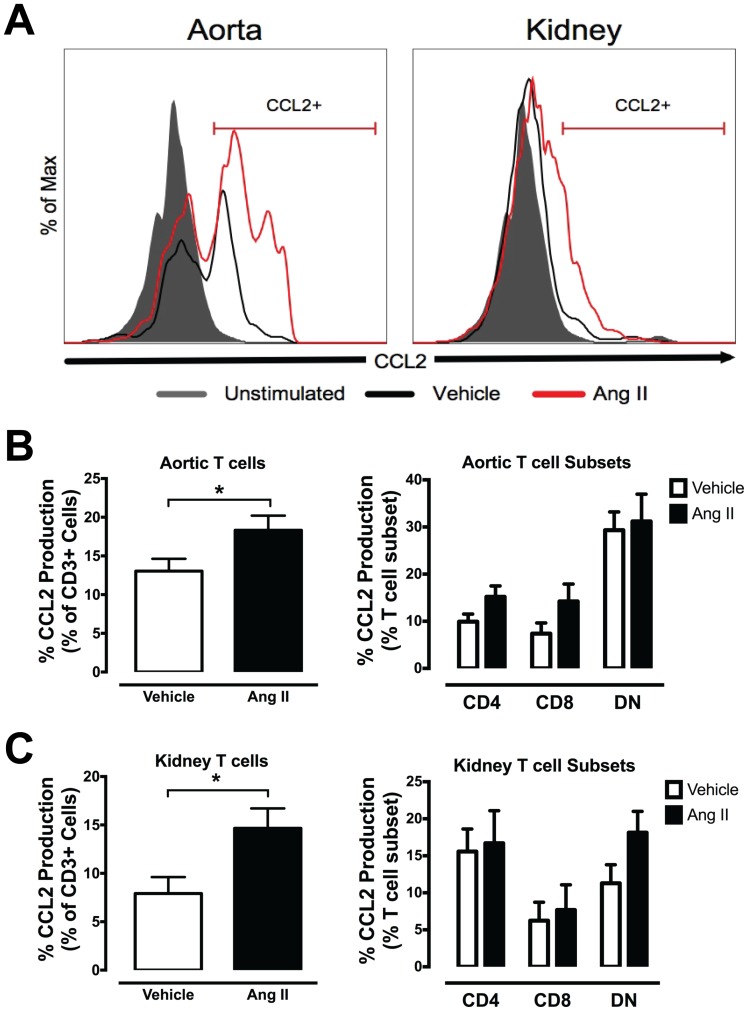
Intracellular FACS analysis of CCL2-producing T cells from aorta and kidney. (A) Representative histograms and gating strategy of CCL2+ T cells from aorta (left) and kidney (right) of vehicle and Ang II-infused mice. (B) Mean data of aortic and (C) kidney T cells (left) and T cell subsets (right) that produce CCL2 in response to PMA-ionomycin stimulation. (*P<0.05 Vs vehicle; Unpaired t test; n = 5–10).

We also examined the proportion of T cells that produced the chemoattractant CCL2 in aorta and kidney. Interestingly, a greater proportion of CCL2-producing aortic and kidney T cells was observed in Ang II-infused mice (Figs. 3A and 3B), which appeared to be derived from CD4+ and CD8+ T cell subsets in the aorta, but DN T cells only in the kidney (Fig. 3B).

### Cytometric Bead Array Analysis of Cytokines and CCL2

**Figure 4:**
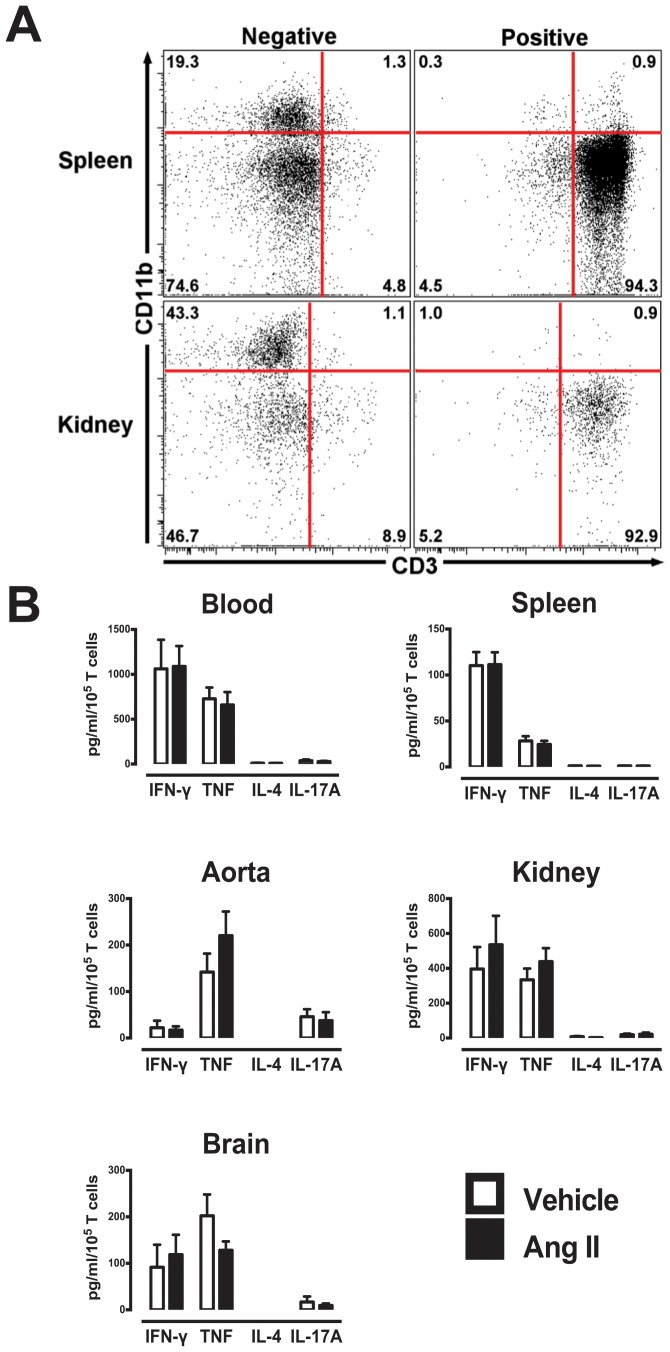
Quantitative cytokine production from isolated T cells from various organs. (A) Purity of T cell (CD3+) isolation. Representative flow cytometry plots of spleen and kidney samples following magnetic bead T cell isolation. Negative samples represent cells remaining after T cells had been extracted, and the positive samples represent extracted cell samples. (B) Mean data of cytokine production after anti-CD3 stimulation using CBA assay. Data represented as total amount of cytokine produced (A) in pg/ml per 10^5^ T cells in blood, spleen, aorta, kidney and brain (n = 7–11).

Following T cell isolation using the CD3+ magnetic microbeads, flow cytometry confirmed greater than 90% of the isolated cells were CD3+ T cells in all organs. CD11b positive cells (myeloid cells including macrophages) represented ≤1% of all leukocytes (Fig. 4A). Consistent with intracellular FACS analysis, a cytometric bead array revealed a predominant Th1 cytokine phenotype was observed from T cells of all organs, but no significant differences in T cell-derived cytokine production following anti-CD3/CD28-stimulation was observed in vehicle- or Ang II-treated mice (Fig. 4B). Blood and kidney T cells appeared to produce more cytokines compared to other organs. T cell-derived IL-6 and IL-10 production was also detected from most organs, however, no differences were observed between T cells from normotensive and hypertensive mice (S4 and S5 Figures).

**Figure 5:**
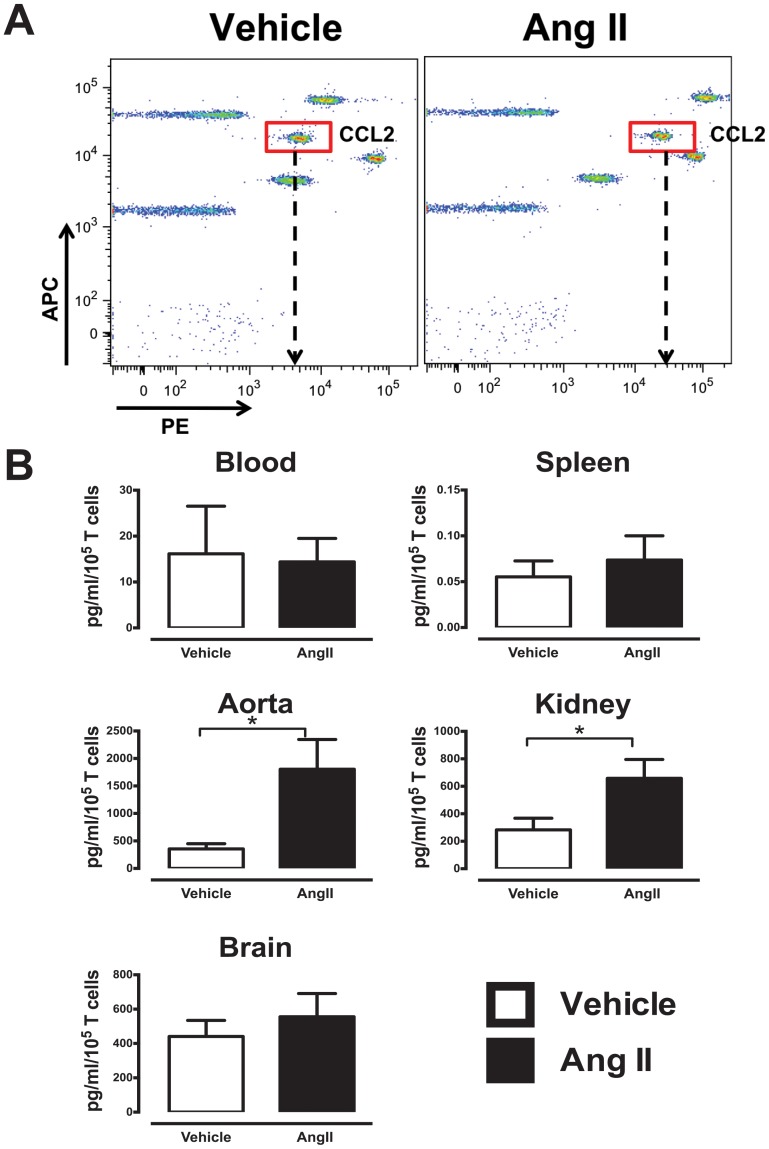
Infiltrating T cells from hypertensive mice produce greater CCL2. (A) Representative CBA assays plot and gating of CCL2 in a vehicle and Ang II aortic sample. Arrow denotes the mean fluorescence intensity of each sample. (B) Quantitative analysis of amount of CCL2 produced using CBA assay. Data represented as total amount of cytokine produced in pg/ml per 10^5^ T cells in blood, spleen, aorta, kidney and brain (n = 11–22). (*P<0.05 Vs vehicle; Unpaired T test; n = 7–8).

The levels of the chemokine, CCL2, following anti-CD3 stimulation were also measured using a CBA (Fig. 5). Interestingly, while there were no differences in T cell-derived CCL2 production from T cells of blood and spleen of vehicle- or Ang II-treated mice, tissue infiltrating T cells of Ang II-treated mice produced significantly greater CCL2 in aorta (∼6 fold) and kidney (∼3 fold). No differences in CCL2 production were observed in brain (Fig. 5B).

### T cell–Derived ROS production

**Figure 6:**
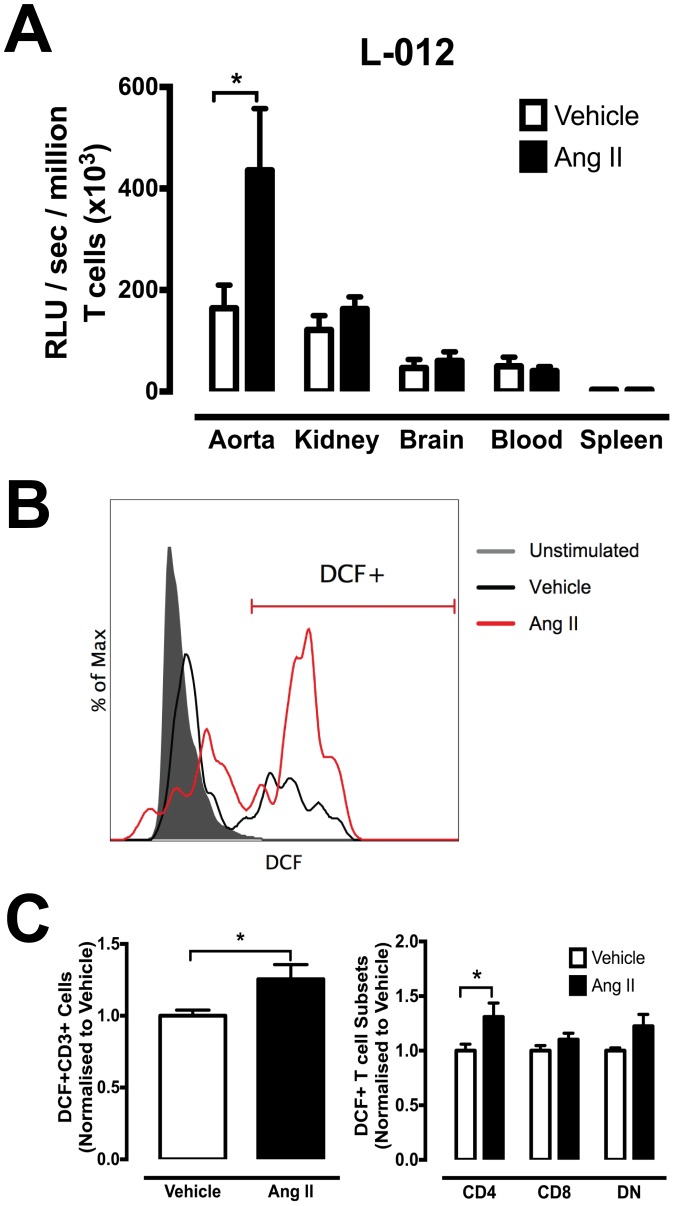
Effect of Ang II-induced hypertension on T cell-derived ROS production. (A) L-012 enhanced chemiluminescence detection of extracellular ROS from various tissue samples from vehicle and Ang II-infused mice. Data represented as the amount of ROS produced in RLU/sec per million T cells (x10^3^) with PDBu stimulation in kidney, brain blood and spleen (*P<0.05 Vs vehicle; n = 7–14). (B) Representative histograms and gating strategy of DCF+T cells from aorta. (C) Mean DCF+T cells normalized to response in corresponding vehicle T cells (left) and T cell subsets (right) (*P<0.05 Vs vehicle; n = 5–11).

L-012 chemiluminescence was used to detect the amount of ROS produced by T cells from various organs. There was no difference in the basal level of T cell-derived ROS between vehicle- and Ang II-treated mice (data not shown). The amount of T cell-derived ROS following PDBu stimulation was significantly elevated in tissue-infiltrating T cells from aorta of Ang II-treated mice (Fig. 6A), but this difference was not observed from T cells of any other organs. Flow cytometric analysis demonstrated a significant increase in PDBu-stimulated intracellular ROS production in aortic T cells from Ang II-infused mice compared to vehicle-treated mice (Fig. 6B and 6C). Interestingly, this increase was only evident in CD4+ and DN T cell subsets within the aorta (Fig. 6C).

## Discussion

The major findings of the present study were that the majority of T cells were of the Th1 pro-inflammatory phenotype and produced relatively the same amount of cytokines in normotensive and hypertensive mice, although there was greater T cell infiltration per se into aorta and kidney in Ang II-infused mice. By contrast, there was enhanced aortic CCL2 and ROS production from each T cell-infiltrate during Ang II-induced hypertension, which may represent a hypertension-specific and unique T cell phenotype that is likely to contribute to hypertension and associated vascular diseases.

Hypertension is now considered an inflammatory and immune system disorder. While it is known that there is increased infiltration of T cells into important blood pressure controlling organs during hypertension, the pathophysiological function of these infiltrating T cells remains unclear. Current literature suggests that one of the major contributions of T cells to hypertension is through the release of cytokines from CD4+ T (helper) cell subsets that promote local inflammation, which under normal physiological conditions would facilitate removal of foreign pathogens [6], [17], [19]. While we and others have reported significant elevations of Th1 cytokines, these observations have been limited to T cells isolated from secondary lymphoid organs [16], [17] and thus no studies have directly examined the cytokine profiles of T cells isolated directly from blood pressure-controlling organs. Using well-established immunological tools to define T cell cytokine profiles, we thoroughly examined T cell-derived cytokine production in response to either anti-CD3 or PMA-ionomycin in various tissues. From our studies, it is clear that for the Th cytokines we have analysed, hypertension is not associated with differences in the proportion of cytokine-producing T cells or the amount of cytokine release per T cell from normotensive and Ang II-infused hypertensive mice. We observed no differences in the amount of cytokines produced from non-infiltrating (blood and spleen) compared to infiltrating T cells. However, there was a predominant Th1 phenotype with greater IFN-γ and TNF-α production, which most likely reflects the C57BL6/J mouse strain [20], [21]. C57BL6/J mice are also more prone to hypertension and associated systemic and vascular inflammation compared to Th2 prone BALB/c mice, which would further support an association between hypertension and the Th1 phenotype [22]. However, inhibition of Th1 cytokines using agents such as the TNF-α antagonist, etanercept, to treat experimental hypertension has been equivocal, and consistent with our findings, it suggests that cytokine production may not be the most important or hypertension-specific mechanistic role for T cells during hypertension[6], [23]–[25]. Our findings however, do not rule out a contribution of cytokines to local inflammation. Consistent with the current literature, we observed greater T cell infiltration into blood pressure-controlling organs such as the aorta, kidney and for the first time we showed in brain that there is increased T cell infiltration during hypertension [6], [26]–[28]. We also observed greater FoxP3+ T regulatory cell infiltration into the aorta of hypertensive mice, which may suggest non-selective recruitment of T cells during hypertension. Although this differs from a previous study that examined aortic T regulatory cell number in Ang II-infused mice, this may be due to the quantitative approach used. Barhoumi and colleagues [29] used immunofluorescence as opposed to flow cytometry in the current study, which is a fully quantitative technique and examined total FoxP3+ cell across the full length of the aorta. Nonetheless, while infiltrating T cells may not produce more pro-inflammatory cytokines during hypertension, greater accumulation of T cells possibly increases the overall cytokine levels locally, thereby promoting inflammation associated with hypertension. A corollary from our findings may be that prevention of the initial T cell infiltration into blood pressure-controlling organs represents a potential therapeutic strategy to negate inflammation associated with hypertension.

We identified three T cell subsets that were distinct during hypertension. We discovered that there were unique subsets that produced more IFN-γ in specific organs in hypertension, including CD8+ cells in aorta, DN cells in kidney and possibly DN cells in blood (Table 1). While their specific role remains unidentified, these are unique phenotypes that are specifically elevated during hypertension. To our knowledge, no studies have examined specific T cell subsets in hypertension; however, unique subsets have been observed in other immune diseases. In the synovial fluid from patients with rheumatoid arthritis, an elevated proportion of IFN-γ producing CD4+ and CD8+ T cells have been reported [30]. Wahlstrom et al [31] have also shown increased IFN-γ- and TNF-α-producing CD4+ and CD8+ T cell subsets in the bronchoalveolar lavage fluid from sarcoidosis patients; findings that have in fact aided further elucidation of the pathogenic mechanisms in sarcoidosis. Therefore, like other immune disorders, identification of these unique subsets in hypertension represents a step forward in the understanding the local pathology of T cells in the setting of hypertension, and warrants further investigation into the function of these phenotypes.

Importantly, we also identified a distinct infiltrating T cell phenotype during hypertension, where a significantly greater proportion of T cells in aorta and kidney produced CCL2, which also translated to greater amounts of CCL2 detected in conditioned media of stimulated T cells. This increase was absent in circulating or splenic T cells, and since there was increased T cell infiltration into these tissues, CCL2 production may be further amplified during hypertension. CCL2 is a vital chemokine that promotes leukocyte recruitment, and elevated production can result in an enhanced local inflammatory response [12], [32], which may lead to advanced inflammation and organ dysfunction resulting in overt hypertension. T cells are known to produce CCL2 [33], [34], although macrophages are also an important source [35]. Consistent with our findings, previous studies have shown increased CCL2 production from infiltrating T cells in neoplasia/cancer [36], [37] and infiltrating leukocytes in arthritis [12], [38]. Our findings suggest that a hypertension-specific function of infiltrating T cells during hypertension may be to promote further leukocyte recruitment via increased CCL2 production. We speculate that recruited leukocytes, such as macrophages, may then further promote inflammation through cytokine/chemokine release in a feed-forward fashion. While elevated CCL2 in blood pressure-controlling organs has been shown [39], to our knowledge this is the first evidence that T cells are an important local source of CCL2 that may constitute a vital contribution to inflammation associated with hypertension. Leukocytes are known to express CCR2, to which CCL2 and several other chemokines such as CCL8, CCL13 and CCL27 can bind. Mice deficient in CCR2 exhibit decreased macrophage and monocyte infiltration into the arterial wall during Ang II-induced hypertension [39], suggesting that the CCR2:CCL2 axis is vital for leukocyte recruitment during hypertension. More recently we reported that CCR2 inhibition reduces vascular macrophage accumulation and reverses pressor responses in DOCA-salt induced hypertensive mice [18]. Thus the CCR2:CCL2 axis is an important pathway during hypertension and associated vascular disease, and may be initially driven by infiltrating T cells leading to sequalae of pro-inflammatory events.

In addition to elevated CCL2 production, a greater proportion of infiltrating T cells from aorta of hypertensive mice were shown to produce higher levels of intracellular and extracellular ROS. It is well established that elevated ROS results in much greater oxidative stress and promotes inflammation and organ dysfunction during hypertension. [40]–[42]. Guzik and colleagues [6] demonstrated that adoptive transfer of T cells can lead to impaired endothelium-dependent vasodilatation and increased ROS production following Ang II infusion in RAG-1^−/−^ mice [6]. Importantly, adoptive transfer of T cells deficient in p47phox (an important subunit of Nox2 and Nox1 oxidase) into RAG-1^−/−^ mice failed to fully restore hypertensive responses to Ang II, highlighting the potential role of T cell-derived ROS in hypertension [6]. Interestingly, this phenotypical difference was only localized to aortic T cells (predominantly CD4+ and DN T cell subsets) and was absent in all T cells from other organs, suggesting distinct regional effects of T cells during hypertension. Indeed it has been documented that T cells can behave differently in the presence of different cells [42]. Infiltrating T cells of the kidney have been suggested to promote oxidative stress and hypertension in Dahl salt-sensitive rats [43]. We did not observe greater T cell-derived ROS from kidney T cells, however, it is highly likely that other immune cells that can be recruited by T cells, such as macrophages or neutrophils, could act as sources of ROS in the kidney. Importantly, there was no change in ROS production in T cells from non-infiltrating organs (blood and spleen), which is consistent with CCL2 effects where an increase in CCL2 production was limited to infiltrate only. Indeed there is functional interplay between ROS and CCL2. ROS are known to increase CCL2 expression and infiltration of inflammatory cells in pressure-overloaded rat hearts [44]. Chen et al [45] reported that inhibition of NADPH oxidase reduced CCL2 mRNA accumulation in vascular endothelial cells, indicating that ROS plays an important role in the modulation of CCL2 gene expression. It is important to note that we employed the Ang II-infusion model of hypertension, and Ang II may directly stimulate the effects we have observed in CCL2 and ROS production. However, based on previous studies, the interplay between T cells and the development of hypertension has been observed in several models of hypertension such as DOCA-salt- and noradrenaline-induced hypertension, which suggests that elevated pressure induces activation of the adaptive immune system. Moreover, DOCA-salt-induced hypertension is also associated with elevated aortic CCL2 mRNA expression [18]. Importantly, most human hypertension is associated with greater plasma levels of Ang II, particularly frequent in malignant hypertension [46], [47]. Thus, infiltrating T cells may be responsible for the functional interplay of oxidative stress and leukocyte chemotaxis and may represent a vital function of T cells during hypertension.

**Figure 7:**
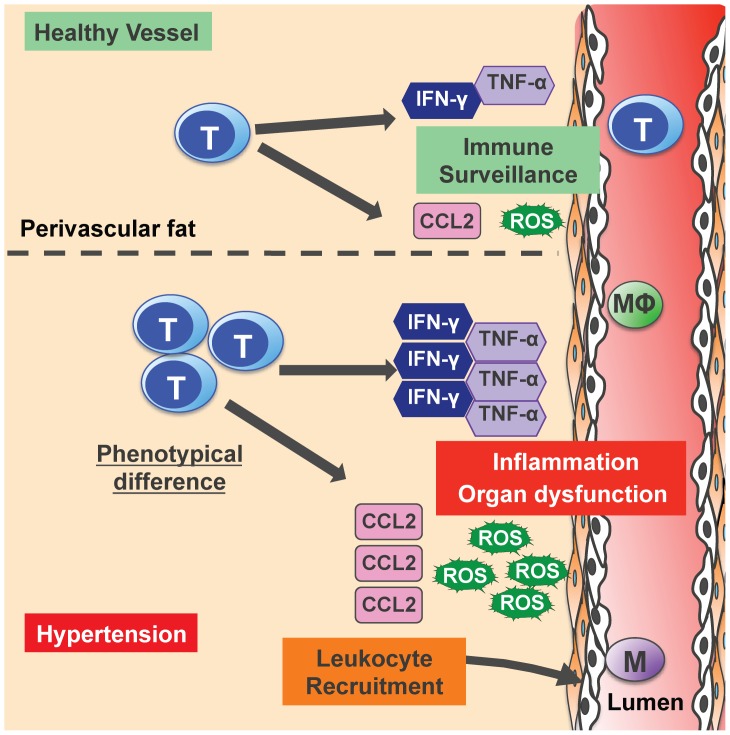
Potential functions of infiltrating T cells during hypertension. Based on the results to our study and current literature, we speculate that in healthy vessels, there is basal level of T cell infiltration that may be required for immune surveillance. During hypertension, there is greater T cell infiltration localized to the perivascular fat of the aorta. Although each T cell infiltrate produces that same amount of cytokines as in healthy vessels, greater T cell infiltrate during hypertension may result in an overall net increase cytokines produced. However, indicative of a phenotypical difference, T cell infiltrates in vessels of hypertensive mice produce greater CCL2 and ROS, which can result in enhanced leukocyte recruitment such as macrophages (MΦ), oxidative stress and local inflammation that can lead to vascular dysfunction and exacerbate hypertension.

Collectively, we have demonstrated that cytokine profiles from infiltrating T cells are not differentially modulated by Ang II-induced hypertension, but rather the local milieu may be influenced by greater accumulation of T cells during hypertension. Moreover, aortic and kidney T cells exhibit greater CCL2 and ROS production (aorta only), and represent a hypertension-specific phenotype that may promote the local inflammatory responses by recruiting leukocytes and causing oxidative stress (Fig. 7). Based on our findings, specifically targeting leukocyte recruitment and homing of T cells to blood pressure controlling organs, may represent a potential therapeutic approach to reduce the burden of inflammation that is associated with hypertension and likely many other related vascular disorders.

## Supporting Information

S1 Figure **Total number of (A) aortic and (B) kidney infiltrating T cell subsets in vehicle and Ang II-infused mice.**
(TIFF)

S2 Figure **Effector phenotype (CD44^hi^+CD62L^lo^) of (A) aortic and (B) kidney infiltrating T cell subsets in vehicle and Ang II-infused mice.** (*P<0.05, ***P<0.001 Vs vehicle; Unpaired t test; n = 6–14).
(TIFF)

S3 Figure **FoxP3+ T regulatory cell infiltration.** (A) Representative gating strategy for FoxP3+ cells (T regulatory cells) in aorta and kidney. After exclusion on dead cells, total leukocytes (CD45+), T cells (CD3+) and T cell subsets (CD4+, CD8+, DN) were sequentially gated. Finally Foxp3+ cells were gated from CD4+ T cells. (B) Mean data of infiltrating FoxP3+ T cells within aorta (left) and kidney (right) from vehicle and Ang II-infused mice. (*P<0.05 Vs vehicle; Unpaired t test; n = 6).
(TIF)

S4 Figure **IL-6 production from blood and organ-isolated T cells.** Quantitative analysis of amount of IL-6 produced following anti-CD3/CD28 stimulation using a CBA. Data represented as total amount of IL-6 produced in pg/ml per 10^5^ T cells in blood, spleen, aorta, kidney and brain (n = 11–22).
(TIFF)

S5 Figure **IL-10 production from blood and organ-isolated T cells.** Quantitative analysis of amount of IL-10 produced following anti-CD3/CD28 stimulation using CBA assay. Data represented as total amount of IL-10 produced in pg/ml per 10^5^ T cells in blood, spleen, aorta and kidney (n = 11–22).
(TIFF)
