# Supplementary figures and images for: Differential Phenotypes of Tissue-Infiltrating T Cells during Angiotensin II-Induced Hypertension in Mice
Source: PLoS One. 2014 Dec 11;9(12):e114895. doi: 10.1371/journal.pone.0114895 (PMC4263711; doi:10.1371/journal.pone.0114895)

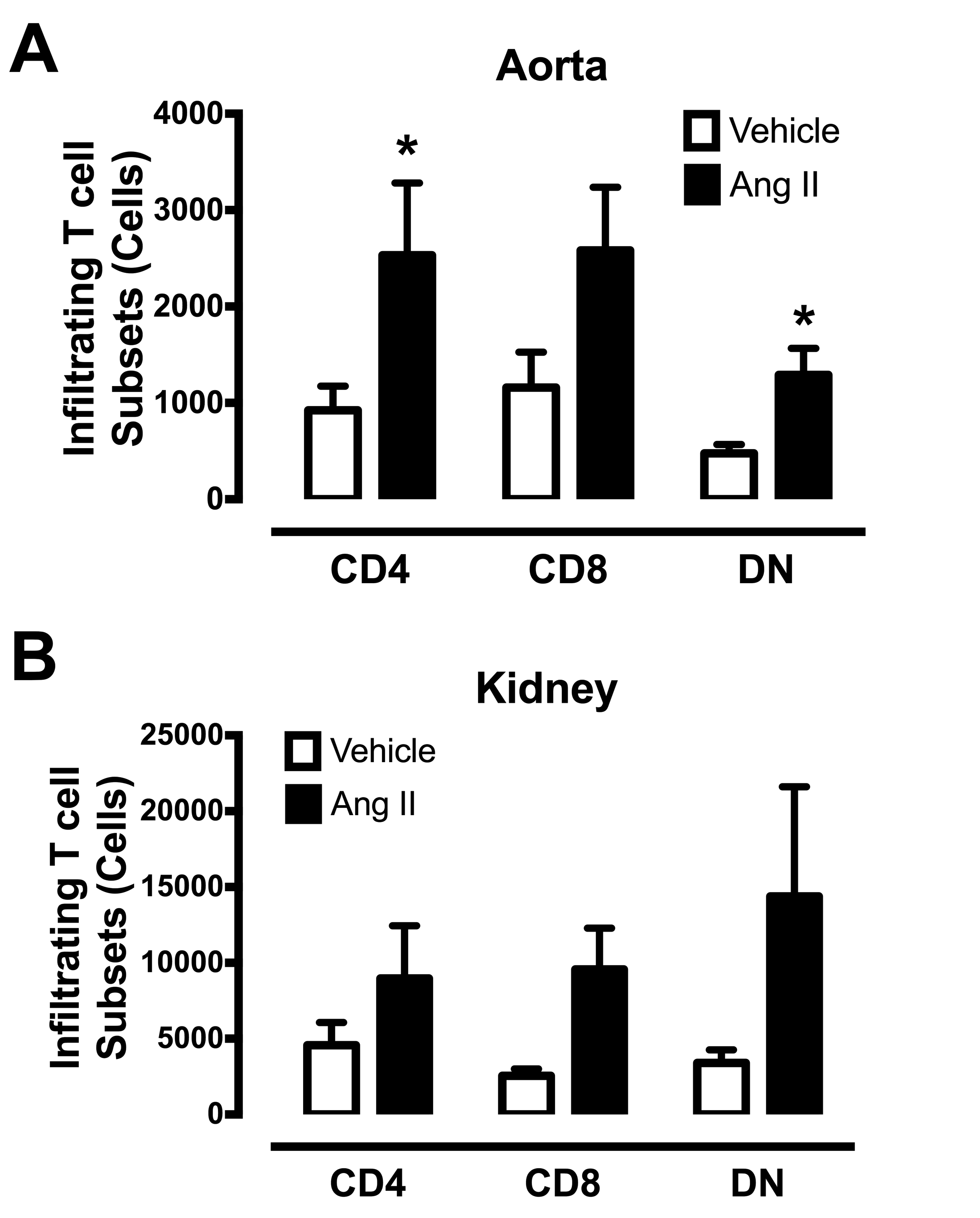

Supplement: S1 Figure — Total number of (A) aortic and (B) kidney infiltrating T cell subsets in vehicle and Ang II-infused mice. (TIFF) [file pone.0114895.s001.tiff]

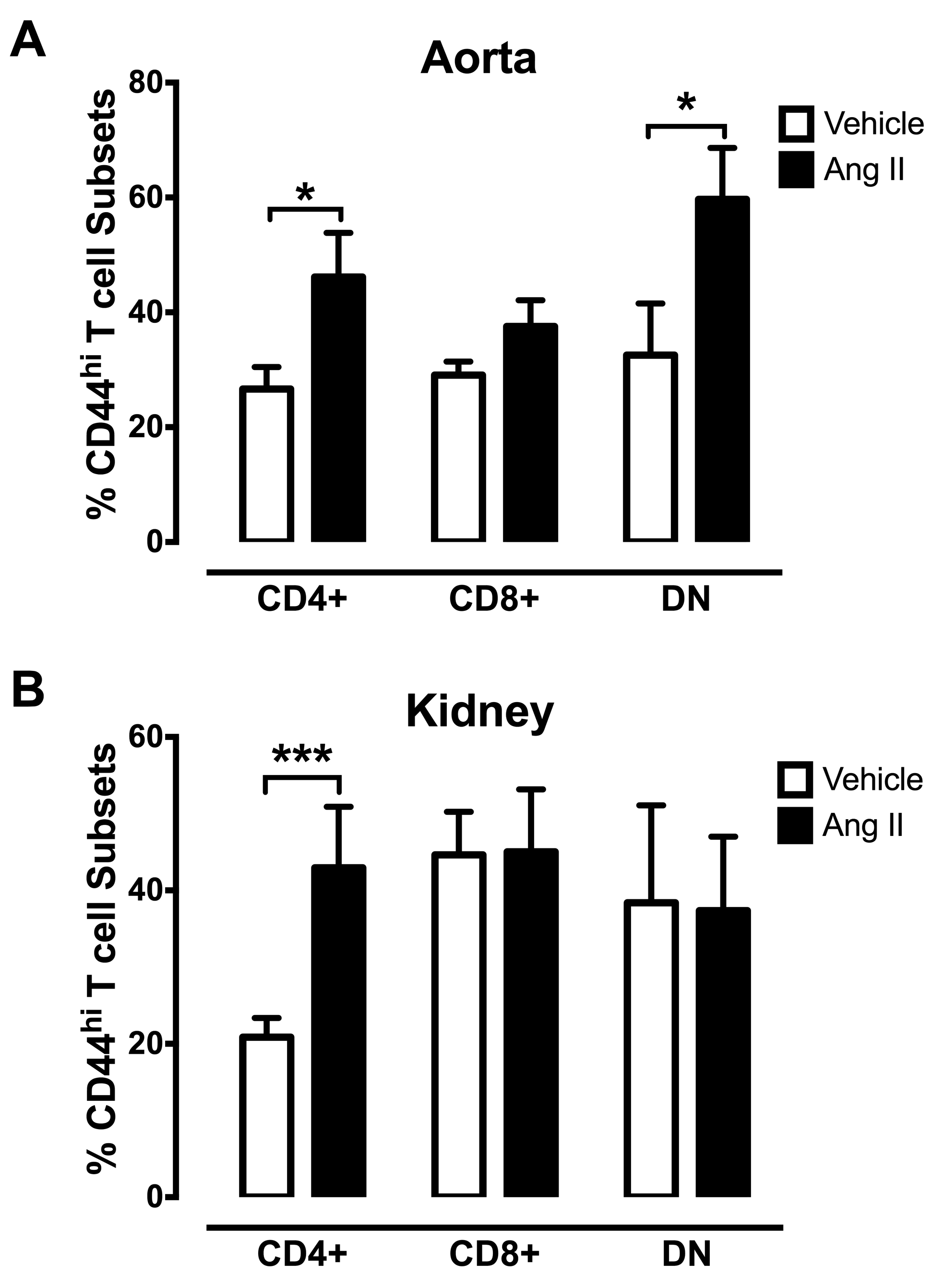

Supplement: S2 Figure — Effector phenotype (CD44hi+CD62Llo) of (A) aortic and (B) kidney infiltrating T cell subsets in vehicle and Ang II-infused mice. (*P<0.05, ***P<0.001 Vs vehicle; Unpaired t test; n = 6–14). (TIFF) [file pone.0114895.s002.tiff]

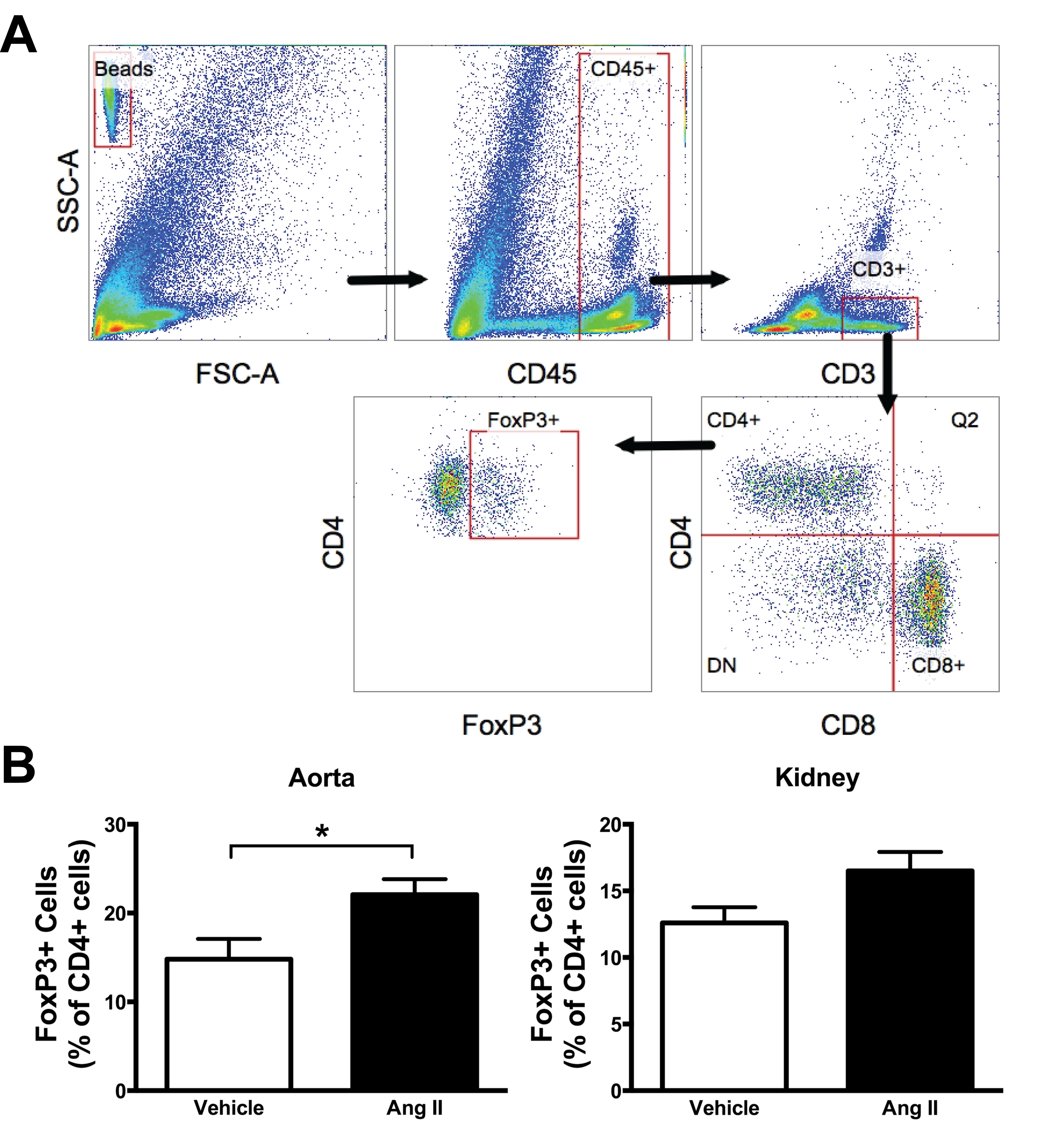

Supplement: S3 Figure — FoxP3+ T regulatory cell infiltration. (A) Representative gating strategy for FoxP3+ cells (T regulatory cells) in aorta and kidney. After exclusion on dead cells, total leukocytes (CD45+), T cells (CD3+) and T cell subsets (CD4+, CD8+, DN) were sequentially gated. Finally Foxp3+ cells were gated from CD4+ T cells. (B) Mean data of infiltrating FoxP3+ T cells within aorta (left) and kidney (right) from vehicle and Ang II-infused mice. (*P<0.05 Vs vehicle; Unpaired t test; n = 6). (TIF) [file pone.0114895.s003.tif]

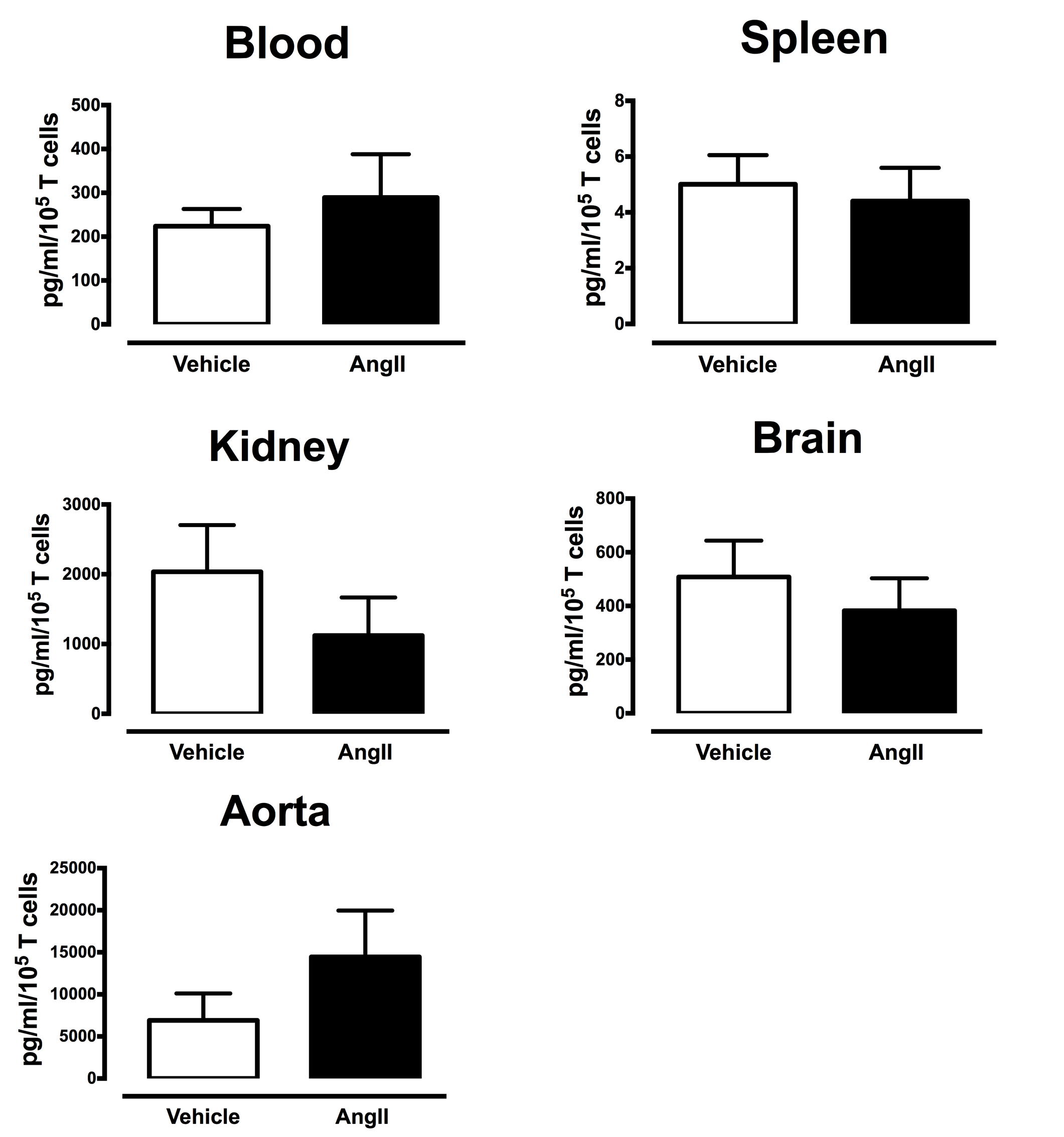

Supplement: S4 Figure — IL-6 production from blood and organ-isolated T cells. Quantitative analysis of amount of IL-6 produced following anti-CD3/CD28 stimulation using a CBA. Data represented as total amount of IL-6 produced in pg/ml per 105 T cells in blood, spleen, aorta, kidney and brain (n = 11–22). (TIFF) [file pone.0114895.s004.tiff]

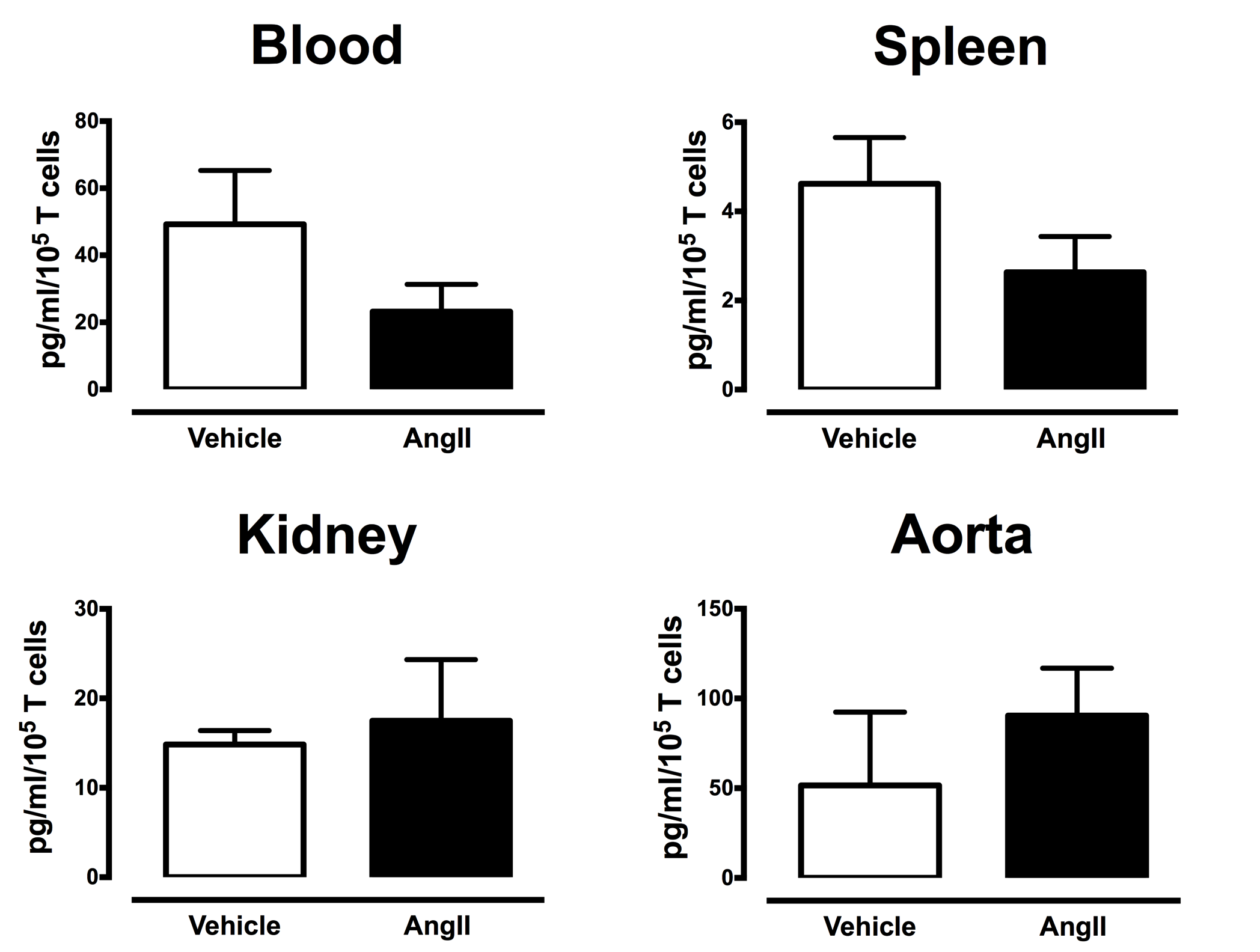

Supplement: S5 Figure — IL-10 production from blood and organ-isolated T cells. Quantitative analysis of amount of IL-10 produced following anti-CD3/CD28 stimulation using CBA assay. Data represented as total amount of IL-10 produced in pg/ml per 105 T cells in blood, spleen, aorta and kidney (n = 11–22). (TIFF) [file pone.0114895.s005.tiff]
